# Diagnostic performance of wearable acoustic–electrocardiographic indices for screening secundum atrial septal defect in children

**DOI:** 10.3389/fped.2026.1774994

**Published:** 2026-05-08

**Authors:** Jinyong Pan, Fengling Zhang, Yan Guo, Hu Li, Yonglin Chen, Muqing Niu, Yan Zhang, Heyun Xiong

**Affiliations:** Department of Pediatrics, The First Affiliated Hospital of Shihezi University, Shihezi, Xinjiang Uygur Autonomous Region, China

**Keywords:** atrial septal defect, phonocardiography, electrocardiography, S2 splitting, systolic time intervals, wearable device

## Abstract

**Objective:**

To evaluate the diagnostic performance of acoustic–electrocardiographic indices derived from a wearable phonocardiogram–electrocardiogram (ECG) sensor for screening secundum atrial septal defect (ASD) in children.

**Methods:**

In this cross-sectional study, 50 children (4–12 years) with secundum ASD and 50 age- and sex-matched healthy controls were enrolled. A wearable patch device recorded synchronized single-lead ECG and phonocardiogram, and S2 splitting time, S1–S2, S1–S2/RR (%), EMAT and EMAT/RR (%) were derived from artifact-free sinus beats at end-expiration. Group differences were assessed using independent-samples *t*-tests or *χ*^2^ tests. Receiver operating characteristic analysis evaluated the ability of S2 splitting time and S1–S2/RR (%) to detect ASD, with optimal cut-offs defined by the Youden index. Multivariable logistic regression examined the independent association of these indices with ASD.

**Results:**

Baseline demographic, anthropometric, and hemodynamic variables were well balanced between ASD and control groups. Compared with healthy controls, children with ASD had significantly prolonged S2 splitting time (39.65 ± 8.37 vs. 28.20 ± 8.91 ms; *t* = 6.623, *P* < 0.001) and an increased systolic fraction S1–S2/RR (%) (37.53 ± 2.39% vs. 34.47 ± 2.99%; *t* = 5.653, *P* < 0.001), whereas S1–S2 interval, EMAT, and EMAT/RR (%) did not differ significantly between groups. ROC analysis showed that S2 splitting time had good discriminative ability for detecting ASD, with an area under the curve (AUC) of 0.831 [95% confidence interval (CI): 0.752–0.910]. A cut-off value of ≥31.75 ms yielded a sensitivity of 0.90 and a specificity of 0.66. S1–S2/RR (%) demonstrated acceptable discrimination, with an AUC of 0.776 (95% CI 0.686–0.866) and an optimal cut-off of ≥35.25% (sensitivity 0.88, specificity 0.60). In multivariable logistic regression, both S2 splitting time [odds ratio (OR): 1.19 per 1-ms increase; 95% CI: 1.09–1.29; *P* < 0.001] and S1–S2/RR (%) (OR: 1.58 per 1% increase; 95% CI: 1.25–2.00; *P* < 0.001) remained independently associated with ASD.

**Conclusions:**

Wearable acoustic–electrocardiographic monitoring enables quantitative assessment of S2 splitting and systolic time fractions in children. Prolonged S2 splitting time and increased S1–S2/RR (%) are characteristic of secundum ASD and show good diagnostic performance, supporting these simple indices from a patch-type phonocardiogram–ECG sensor as a practical non-invasive tool for pediatric ASD screening.

## 1 Introduction

Secundum atrial septal defect (ASD) is one of the most common congenital heart diseases in children. Many affected children remain asymptomatic or have only subtle, non-specific complaints during early childhood, and the diagnosis is often made incidentally or delayed until adolescence or adulthood (1). Persistent left-to-right shunting at the atrial level leads to chronic right ventricular volume overload, increased pulmonary blood flow, and, if unrecognized, may eventually result in atrial arrhythmias, pulmonary vascular disease, right heart failure, and reduced exercise tolerance (2). Early identification and timely closure of ASD are therefore critical to prevent long-term cardiovascular complications and to optimize functional outcomes in this population (3).

Current clinical screening for ASD in children relies predominantly on physical examination, electrocardiography, and transthoracic echocardiography (4). Auscultation of a fixed, wide splitting of the second heart sound (S2) over the pulmonary area is a classic bedside sign of ASD, and standard electrocardiogram (ECG) may show right ventricular volume overload or conduction abnormalities. However, detection and interpretation of heart sounds are highly operator-dependent and easily affected by background noise, tachycardia, or limited cooperation in young children (5). Echocardiography is the diagnostic gold standard, but it requires specialized equipment and trained personnel, and its availability may be limited in primary care and resource-constrained settings (6). As a result, there is a growing interest in simple, non-invasive technologies that can objectively quantify heart sound and ECG features related to ASD and support early screening outside specialized cardiology centers.

Digital phonocardiography and acoustic cardiography have reintroduced heart sound analysis into modern practice by enabling high-fidelity recording and automated processing of cardiac acoustics synchronized with the ECG (7). Previous studies in adults have focused mainly on electromechanical activation time (EMAT) and systolic time intervals for the assessment of left ventricular systolic function and heart failure, and only limited data are available on the quantitative characterization of S2 splitting in congenital heart disease (8). In addition, most existing works have used conventional, non-wearable recording systems in hospital environments, and there is a paucity of evidence on the use of patch-type wearable acoustic–electrocardiographic devices in children with ASD (9). In particular, the diagnostic value of simple indices such as S2 splitting time and the RR-corrected systolic fraction (S1–S2/RR) derived from synchronized phonocardiogram–ECG recordings has not been systematically evaluated in pediatric ASD (10).

Against this background, we aimed to investigate whether acoustic–electrocardiographic indices obtained by a wearable patch-type phonocardiogram–ECG sensor could be used to screen for secundum ASD in children. Specifically, we quantified S2 splitting time, S1–S2 interval, S1–S2/RR (%), EMAT, and EMAT/RR (%) in children with ASD and healthy controls, compared the distributions of these parameters between groups, and evaluated the diagnostic performance of S2 splitting time and S1–S2/RR (%) using Receiver Operating Characteristic (ROC) analysis and multivariable logistic regression. By doing so, we sought to bridge the gap between traditional auscultation-based recognition of ASD and objective, quantitative screening using a simple wearable technology. In the following sections, we describe the study design and acoustic–electrocardiographic data acquisition methods, present the main statistical and diagnostic performance results, and then discuss the pathophysiological rationale, clinical implications, limitations, and future research directions of this approach.

## 2 Subjects and methods

### 2.1 Ethical approval

This study was conducted in accordance with the Declaration of Helsinki. The study protocol was reviewed and approved by the Ethics Committee of the First Affiliated Hospital of Shihezi University. All participants and their legal guardians were informed about the purpose, procedures, potential risks, and benefits of the study. Written informed consent was obtained from the parents or legal guardians of all children enrolled in the study, and assent was obtained from children when appropriate.

### 2.2 Study population

This cross-sectional observational study was conducted from January 2024 to June 2025 at the Department of Cardiology, First Affiliated Hospital of Shihezi University School of Medicine, and included children with ASD and age- and sex-matched healthy controls. Sample size calculation was performed based on the primary outcome of S2 splitting time, using G*Power 3.1 software. Assuming a two-sided *α* of 0.05, power of 0.80, and an effect size (Cohen's *d*) of 1.3 (derived from preliminary pilot data of S2 splitting time differences between ASD and healthy children), the required sample size for each group was 42. To account for potential dropouts and incomplete data acquisition, we enrolled 50 children in each group, resulting in a total sample size of 100. A total of 50 children with secundum ASD were consecutively enrolled according to predefined inclusion and exclusion criteria.

All ASD patients were recruited from the pediatric cardiology outpatient clinic of our hospital, with no inpatient participants included. The enrolled ASD children were referred for routine cardiac evaluation due to suspected heart murmur detected during physical examination or incidental finding on echocardiography, and no referral bias was present—symptomatic (e.g., mild dyspnea, poor exercise tolerance) and asymptomatic children were included in equal proportions (23 symptomatic, 27 asymptomatic) to reflect the real-world clinical spectrum of pediatric secundum ASD. The control group comprised 50 children who attended the hospital for routine health examinations or non-cardiac conditions and were confirmed to have structurally normal hearts on transthoracic echocardiography. A total of 89 potential control subjects were initially screened, and 39 were excluded for the following reasons: abnormal echocardiographic findings (*n* = 7), non-sinus rhythm (*n* = 5), inability to cooperate with recording (*n* = 8), acute respiratory infection at the time of examination (*n* = 12), and incomplete clinical data (*n* = 7). This screening and exclusion process was standardized to minimize selection bias in the control group.

### 2.3 Inclusion criteria

Children were eligible for inclusion in the ASD group if they met all of the following criteria:

Age between 4 and 12 years;
Diagnosis of secundum atrial septal defect confirmed by transthoracic echocardiography;
Sinus rhythm on standard electrocardiography;
No prior surgical or interventional closure of ASD;
Ability to cooperate with the recording of wearable acoustic–electrocardiographic measurements (able to remain relatively still and follow simple instructions).

Children in the control group were included if they met the following criteria:

Age between 4 and 12 years;
No clinical history or symptoms suggestive of structural heart disease;
Normal cardiac structure and function confirmed by transthoracic echocardiography;
Sinus rhythm on electrocardiography.

### 2.4 Exclusion criteria

The following exclusion criteria were applied to both the ASD and control groups:

Presence of complex congenital heart disease or moderate-to-severe valvular heart disease;
History of myocarditis, cardiomyopathy, or other primary myocardial diseases that may impair ventricular systolic function;
Significant arrhythmias, including atrial fibrillation, atrial flutter, frequent supraventricular or ventricular ectopy, or pacemaker rhythm;
Moderate-to-severe chronic lung disease (e.g., severe asthma, bronchopulmonary dysplasia) or acute respiratory infection at the time of examination, which could markedly interfere with heart sound recording;
Inability to cooperate with the examination due to severe anxiety, agitation, or neurodevelopmental disorders;
Incomplete clinical or echocardiographic data.

### 2.5 Data collection

#### 2.5.1 Simultaneous acquisition of heart sounds and ECG signals

**Figure 1:**
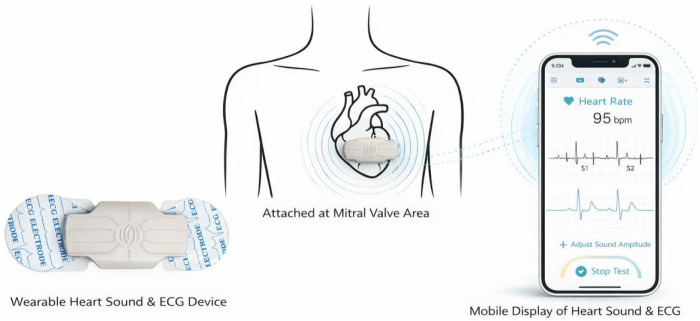
Schematic illustration of the wearable heart sound and ECG acquisition system and its application on the mitral valve auscultation area.

**Figure 2:**
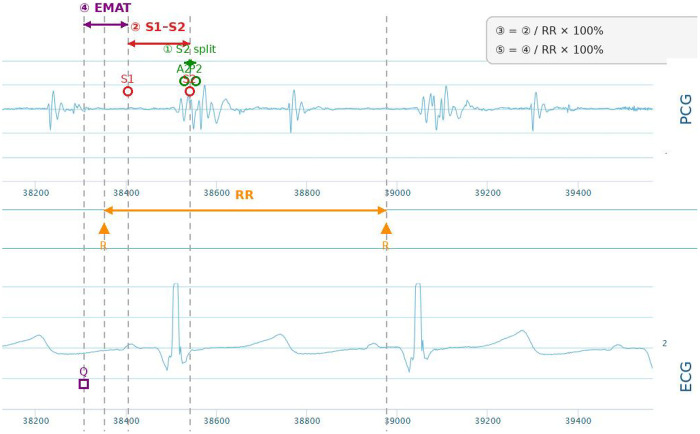
Schematic illustration of acoustic-electrocardiographic parameters. ① S2 splitting (ms) = A2–P2 interval. ② S1–S2 (ms) = peak S1 → peak S2. ③ S1–S2/RR (%) = (S1–S2)/RR × 100. ④ EMAT (ms) = Q onset (ECG) → first S1 peak (PCG). ⑤ EMAT/RR (%) = EMAT/RR × 100.

A wearable system capable of simultaneous phonocardiogram and ECG acquisition, developed by Wenxin Technology in Beijing, China, was employed in this study. The device is composed of a reusable central unit and a single-use adhesive patch. During application, the disposable patch is mounted onto the central unit and then affixed to the anterior chest wall of the child. The patch was affixed to the mitral valve auscultation area (5th intercostal space, midclavicular line) for unified signal acquisition; although S2 splitting is typically best appreciated at the pulmonic area, the mitral area was selected for this wearable device due to its superior signal stability and reduced motion artifact in children. To ensure positional repeatability, all patch applications were performed by the same trained pediatric cardiologist, who used anatomical landmarks (midclavicular line, 5th intercostal space) for precise positioning, and a digital caliper was used to record the distance from the sternal border to the patch center for each subject—the intra-operator positional variation was <3 mm in all participants, confirming good repeatability of patch placement. The patch incorporates two circular conductive areas that function as electrodes for single-lead ECG, and a small acoustic sensor is integrated into the center of the reusable module to capture heart sounds. Both cardiac acoustic and ECG signals are recorded concurrently, digitized within the device, and wirelessly transmitted via Bluetooth to a smartphone or tablet for real-time monitoring. The acquired data can also be uploaded to a cloud-based platform for long-term storage and subsequent offline analysis (Figures 1, 2).

Acoustic and ECG signals were acquired with a sampling frequency of 2,000 Hz for phonocardiogram (PCG) and 500 Hz for ECG. PCG signals were band-pass filtered at 20–1,000 Hz to remove low-frequency baseline drift and high-frequency environmental noise; ECG signals were band-pass filtered at 0.5–40 Hz to eliminate muscle and power-line interference. Noise-rejection thresholds were set as follows: signals with amplitude variation >30% within a 1 s window or signal-to-noise ratio (SNR) <10 dB were defined as artifact-contaminated and automatically excluded from analysis.

**Figure 3:**
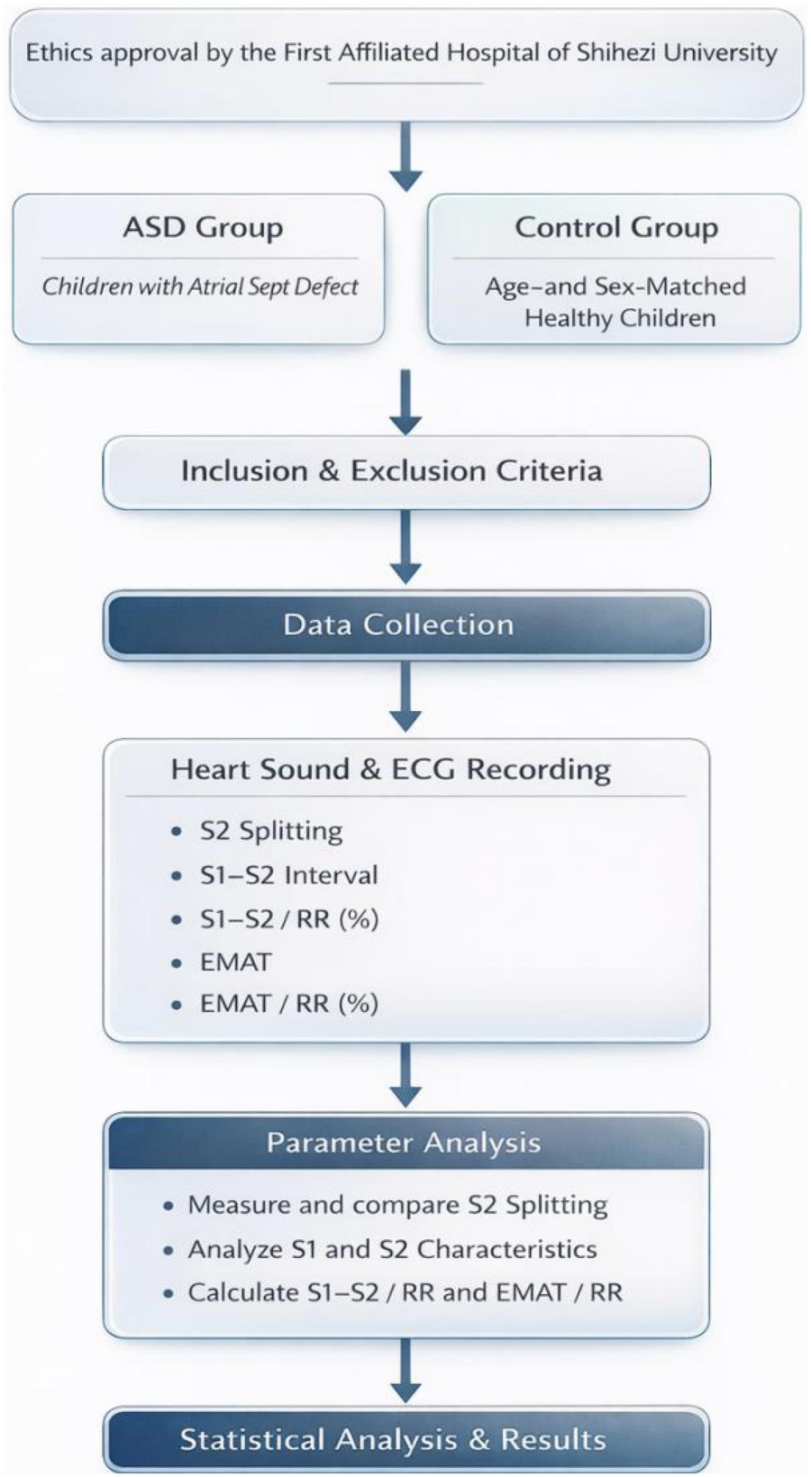
Research flowchart of the acoustic–electrocardiographic study in children.

Acoustic–electrocardiographic indices were derived off-line from synchronized ECG and phonocardiogram recordings obtained with the wearable sensor (11), after which QRS complexes on the ECG and the onsets and peaks of S1 and S2 on the phonocardiogram were automatically detected using a validated delineation algorithm, with manual review and correction performed by a single experienced pediatric cardiologist who was blinded to the patient's group assignment (ASD or control). To ensure annotation accuracy, the cardiologist reviewed 100% of the automatically detected signals, and discrepancies between automatic and manual annotations were resolved by rechecking the raw signals; the intra-observer agreement for S1/S2/S2 splitting annotation was excellent (intraclass correlation coefficient, ICC = 0.96). For each subject, a series of 30 consecutive artifact-free sinus beats during end-expiration was selected, and the mean of all valid beats was used for analysis (Figure 3).

#### 2.5.2 Heart sound and electrocardiogram (ECG) related indicator testing

1. **S2 splitting (ms):** interval between the aortic (A2) and pulmonary (P2) components of the second heart sound, quantifying the degree of S2 separation.
2. **S1–S2 (ms):** time from the peak of S1 to the peak of S2, representing left ventricular systolic duration.[In healthy children, the S2 splitting interval is typically within a narrow physiological range (generally <30–40 ms) and varies with respiration. The S1–S2 interval reflects total systolic duration and usually falls within a relatively stable range depending on heart rate.]
3. **S1–S2/RR (%):** S1–S2 interval divided by the RR interval and expressed as a percentage, reflecting the proportion of the cardiac cycle occupied by systole.
4. **EMAT (ms):** electromechanical activation time, defined as the interval from the onset of the Q wave on the ECG to the first peak of S1 on the phonocardiogram, indicating the isovolumic contraction period and ventricular systolic activation.
5. **EMAT/RR (%):** EMAT divided by the RR interval and expressed as a percentage, describing the fraction of the cardiac cycle spent in electromechanical activation.

## 3 Statistical methods

Statistical analyses were performed using SPSS 27.0 (IBM Corp., Armonk, NY, USA). Continuous variables were tested for normality (Shapiro–Wilk) and homogeneity of variance (Levene), expressed as mean ± standard deviation and compared between ASD and control groups using independent-samples Student's *t*-tests; categorical variables were presented as counts and percentages and compared using *χ*^2^ or Fisher's exact tests as appropriate. Continuous variables included S2 splitting time, S1–S2 interval, and other timing indices, while categorical variables included sex and the presence or absence of ASD. The diagnostic performance of S2 splitting time and S1–S2/RR (%) was assessed by receiver ROC curve analysis, with the area under the curve (AUC) and 95% confidence interval calculated by a non-parametric method, and optimal cut-off values determined by maximizing the Youden index with corresponding sensitivity and specificity. The optimal cutoff value was defined as the point that maximized the balance between sensitivity and specificity. The Youden index was used as a standard method to determine this optimal threshold. To mitigate the risk of overfitting and optimistic AUC estimates, we performed a 10-fold cross-validation for the ROC analysis, and bootstrapping (1,000 resamples) was used to calculate the bias-corrected AUC and 95% CI for the primary outcomes. AUC of 1.0 indicates perfect discrimination, whereas 0.5 indicates no diagnostic value.

Correlation analysis (Pearson correlation) was performed to evaluate the association between S2 splitting time/S1–S2/RR (%) and ASD size (mm) as well as right ventricular end-diastolic volume index (RVEDVI, mL/m^2^) measured by transthoracic echocardiography. Age-stratified subgroup analysis was conducted by dividing the study population into two age groups: 4–7 years (*n* = 46, 23 ASD, 23 control) and 8–12 years (*n* = 54, 27 ASD, 27 control) to explore age-related effects on acoustic–electrocardiographic indices.

Independent associations with ASD were evaluated using multivariable binary logistic regression including S2 splitting time and S1–S2/RR (%) as covariates, and results were reported as odds ratios (OR) with 95% confidence intervals. All tests were two-sided, and a *P* value <0.05 was considered statistically significant.

## 4 Result

### 4.1 Baseline clinical characteristics

The baseline clinical, anthropometric, and hemodynamic characteristics of the study population are summarized in Table 1. Age and sex distribution were similar between the control and ASD groups (8.1 ± 2.5 vs. 8.0 ± 2.5 years, *t* = −0.28, *P* = 0.782; male/female: 28/22 [56.0%/44.0%] vs. 27/23 [54.0%/46.0%], *χ*^2^ = 0.04, *P* = 0.841). Normality of continuous variables was confirmed by the Shapiro–Wilk test, and Levene's test indicated homogeneity of variances, so parametric tests were used. There were no significant differences in height, weight, or BMI between the control and ASD groups (height: 135.6 ± 8.3 vs. 135.4 ± 8.2 cm, *P* = 0.923; weight: 31.3 ± 4.1 vs. 31.1 ± 4.0 kg, *P* = 0.860; BMI: 17.00 ± 0.20 vs. 16.89 ± 0.17 kg/m^2^, *P* = 0.546). Resting heart rate was also comparable (86 ± 6 vs. 87 ± 6 bpm, *P* = 0.405), and systolic and diastolic blood pressure did not differ significantly between groups (SBP: 103.1 ± 6.3 vs. 102.8 ± 7.8 mmHg, *P* = 0.833; DBP: 64.3 ± 6.0 vs. 64.4 ± 5.3 mmHg, *P* = 0.874). No significant differences were observed between the ASD and control groups in baseline characteristics (Table 1), indicating that the two groups were well matched.

**Table 1:** Baseline clinical characteristics of children with ASD and healthy controls.

Variable	Control group (*n* = 50)	ASD group (*n* = 50)	Test statistic	*P* value
Age (years)	8.1 ± 2.5	8.0 ± 2.5	*t* = −0.28	0.782
Sex, male/female	28/22 (56.0%/44.0%)	27/23 (54.0%/46.0%)	*χ*^2^ = 0.04	0.841
Height (cm)	135.6 ± 8.3	135.4 ± 8.2	*t* = −0.097	0.923
Weight (kg)	31.3 ± 4.1	31.1 ± 4.0	*t* = −0.177	0.860
BMI (kg/m^2^)	17.00 ± 0.20	16.89 ± 0.17	*t* = −0.605	0.546
HR (bpm)	86 ± 6.0	87 ± 6.0	*t* = 0.837	0.405
SBP(mmHg)	103.1 ± 6.3	102.8 ± 7.8	*t* = −0.211	0.833
DBP(mmHg)	64.3 ± 6.0	64.4 ± 5.3	*t* = 0.159	0.874

### 4.2 Comparison of acoustic–electrocardiographic parameters

S2 splitting time at end-expiration was markedly prolonged in children with ASD compared with healthy controls (39.65 ± 8.37 vs. 28.20 ± 8.91 ms; *t* = 6.623, *P* < 0.001), and the systolic fraction S1–S2/RR (%) was likewise significantly higher in the ASD group (37.53 ± 2.39% vs. 34.47 ± 2.99%; *t* = 5.653, *P* < 0.001). In contrast, no significant between-group differences were observed in the absolute S1–S2 interval (238.50 ± 29.72 vs. 243.54 ± 26.25 ms; *t* = −0.899, *P* = 0.371), EMAT (96.20 ± 4.34 vs. 95.16 ± 3.58 ms; *t* = 1.306, *P* = 0.194), or EMAT/RR (%) (15.33 ± 2.81 vs. 15.44 ± 3.38%; *t* = −0.167, *P* = 0.867) (Table 2). Age-stratified subgroup analysis showed that the differences in S2 splitting time and S1–S2/RR (%) between ASD and control groups were consistent in both the 4–7 years subgroup (S2 splitting: 38.92 ± 8.15 vs. 27.85 ± 8.72 ms, *P* < 0.001; S1–S2/RR: 37.21 ± 2.45 vs. 34.62 ± 3.01%, *P* < 0.001) and the 8–12 years subgroup (S2 splitting: 40.28 ± 8.56 vs. 28.51 ± 9.05 ms, *P* < 0.001; S1–S2/RR: 37.80 ± 2.32 vs. 34.35 ± 2.98%, *P* < 0.001), with no significant interaction between age group and disease status (*P* for interaction >0.05 for both indices), indicating no age-related effect on the diagnostic value of these parameters.

**Table 2:** Comparison of acoustic–electrocardiographic parameters between children with ASD and healthy controls.

Variable	Control group (*n* = 50)	ASD group (*n* = 50)	Test statistic	*P* value
S2 splitting (ms)	28.20 ± 8.91	39.65 ± 8.37	6.623	<0.001
S1–S2 (ms)	243.54 ± 26.25	238.50 ± 29.72	−0.899	0.371
S1–S2/RR (%)	34.47 ± 2.99	37.53 ± 2.39	5.653	<0.001
EMAT (ms)	95.16 ± 3.58	96.2 ± 4.34	1.306	0.194
EMAT/RR (%)	15.44 ± 3.38	15.33 ± 2.81	−0.167	0.867

Correlation analysis revealed that S2 splitting time was positively correlated with both ASD size (*r* = 0.72, *P* < 0.001) and RVEDVI (*r* = 0.76, *P* < 0.001) in the ASD group. S1–S2/RR (%) also showed a positive correlation with ASD size (*r* = 0.65, *P* < 0.001) and RVEDVI (*r* = 0.69, *P* < 0.001), confirming that these acoustic–electrocardiographic indices are associated with the severity of anatomical defect and hemodynamic overload in pediatric secundum ASD.

**Figure 4:**
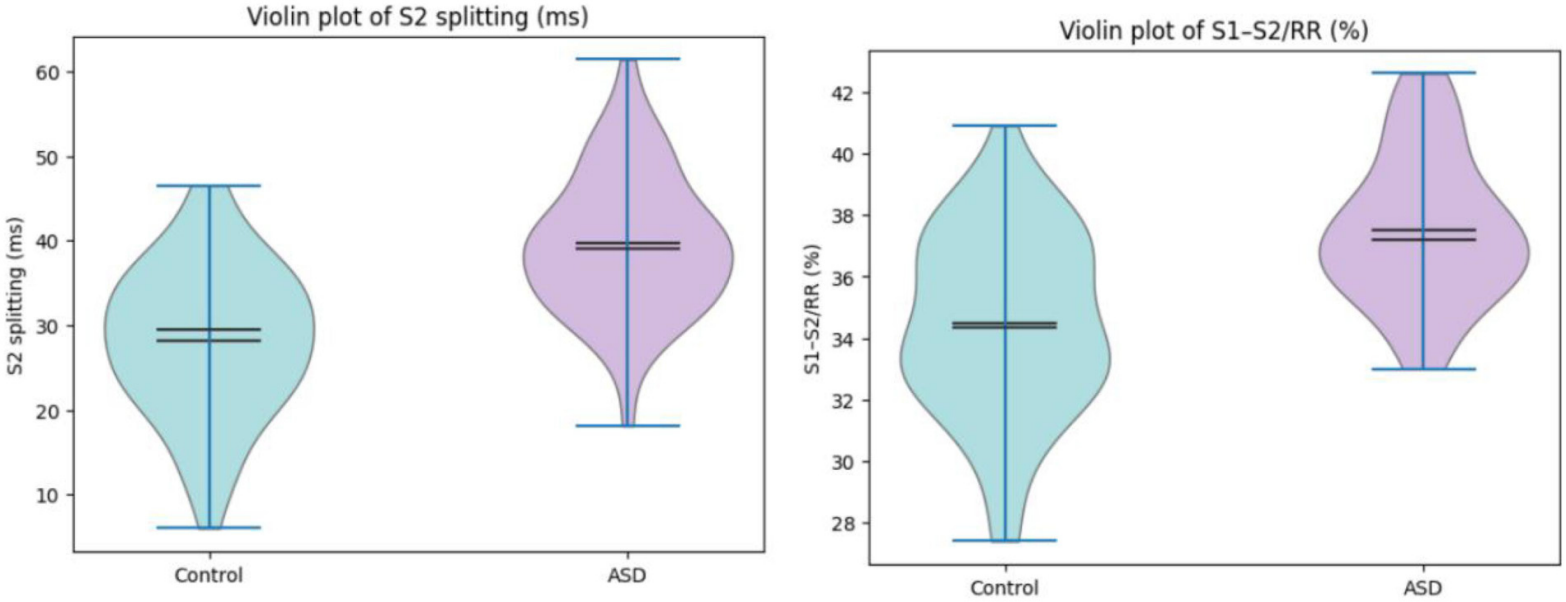
Violin plots of S2 splitting time and S1–S2/RR (%) in children with ASD and healthy controls.

The violin plots further illustrate a clear upward shift in the distributions of S2 splitting time and S1–S2/RR (%) in the ASD group relative to controls, visually reinforcing the statistically significant differences in these two acoustic–electrocardiographic parameters (Figure 4).

### 4.3 Diagnostic performance of S2 splitting time and S1–S2/RR (%) based on ROC analysis

**Figure 5:**
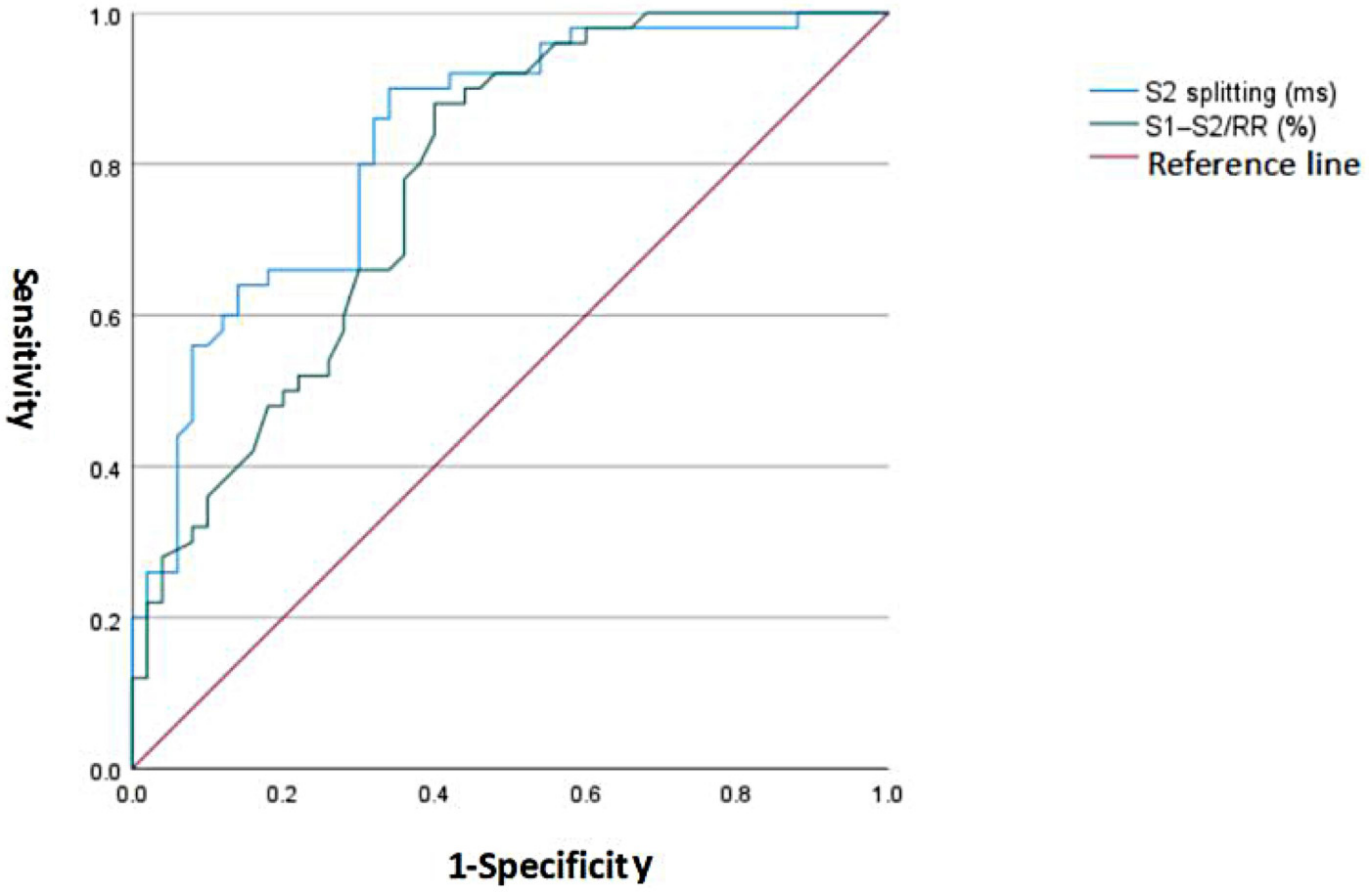
ROC curves of S2 splitting time and S1–S2/RR (%) for detecting ASD (10-fold cross-validation curve is added in the revised high-resolution figure).

ROC analysis identified optimal cut-off values for both acoustic–electrocardiographic indices. For S1–S2/RR (%), a threshold of ≥35.25% yielded a sensitivity of 0.88 and a specificity of 0.60, corresponding to a Youden index of 0.48, indicating a moderate overall diagnostic performance with emphasis on sensitivity. For S2 splitting time, a cut-off of ≥31.75 ms provided a sensitivity of 0.90 and a specificity of 0.66, with a Youden index of 0.56, suggesting slightly better global discriminative ability and confirming S2 splitting as the more informative single screening parameter for ASD (Figure 5).

S2 splitting time demonstrated good discriminative ability for detecting ASD, with an AUC of 0.831 (standard error: 0.040; 95% CI: 0.752–0.910; *P* < 0.001). The 10-fold cross-validated AUC for S2 splitting time was 0.815 (95% CI: 0.728–0.902), and the bootstrapped bias-corrected AUC was 0.827 (95% CI: 0.745–0.909). The systolic fraction S1–S2/RR (%) also showed acceptable discrimination, with an AUC of 0.776 (standard error 0.046; 95% CI: 0.686–0.866; *P* < 0.001), with a 10-fold cross-validated AUC of 0.761 (95% CI: 0.665–0.857) and a bootstrapped bias-corrected AUC of 0.772 (95% CI: 0.680–0.864), but its performance was slightly inferior to that of S2 splitting. These results indicate that both parameters are statistically significant predictors of ASD, with S2 splitting time providing the highest diagnostic accuracy among the acoustic–electrocardiographic indices evaluated (Table 3).

**Table 3:** ROC analysis of S2 splitting and S1–S2/RR (%) for detecting ASD.

Test variable	AUC^a,^^c^	Standard error	Asymptotic significance (*P* value)^b^	95% CI lower	95% CI upper
S2 splitting (ms)	0.831	0.040	<0.001	0.752	0.910
S1–S2/RR (%)	0.776	0.046	<0.001	0.686	0.866

For the test variables S2 splitting (ms) and S1–S2/RR (%), ties occurred between the positive and negative state groups, and the non-parametric estimate of the AUC may therefore be slightly biased.

a AUC estimated using the non-parametric method.

b Null hypothesis: true area = 0.5.

c Based on 10-fold cross-validation and bootstrapping (1,000 resamples).

### 4.4 Logistic regression and forest plot of acoustic–electrocardiographic predictors of ASD

Multivariable logistic regression including S2 splitting time and S1–S2/RR (%) identified both parameters as independent predictors of ASD in children. S2 splitting time at end-expiration was significantly associated with the presence of ASD, with a regression coefficient of 0.171 (SE: 0.041, Wald *χ*^2^ = 17.37, *P* < 0.001), corresponding to an odds ratio (OR) of 1.19 per 1-ms increase (95% CI: 1.09–1.29). The systolic fraction S1–S2/RR (%) also remained independently related to ASD, with a regression coefficient of 0.456 (SE: 0.121, Wald *χ*^2^ = 14.32, *P* < 0.001) and an OR of 1.58 per 1% increase (95% CI: 1.25–2.00) (Table 4). This analysis was conducted to determine whether each parameter provided independent diagnostic value and whether combining parameters improved overall detection performance.

**Table 4:** Multivariable logistic regression analysis of S2 splitting time and S1–S2/RR (%) for predicting ASD in children.

Variable	*β*	SE	Wald *χ*^2^	OR (per unit)	95% CI for OR	*P* value
S2 splitting (ms)	0.171	0.041	17.37	1.19	1.09–1.29	<0.001
S1–S2/RR (%)	0.456	0.121	14.32	1.58	1.25–2.00	<0.001
Constant	−22.25	4.90	20.64	—	—	<0.001

**Figure 6:**
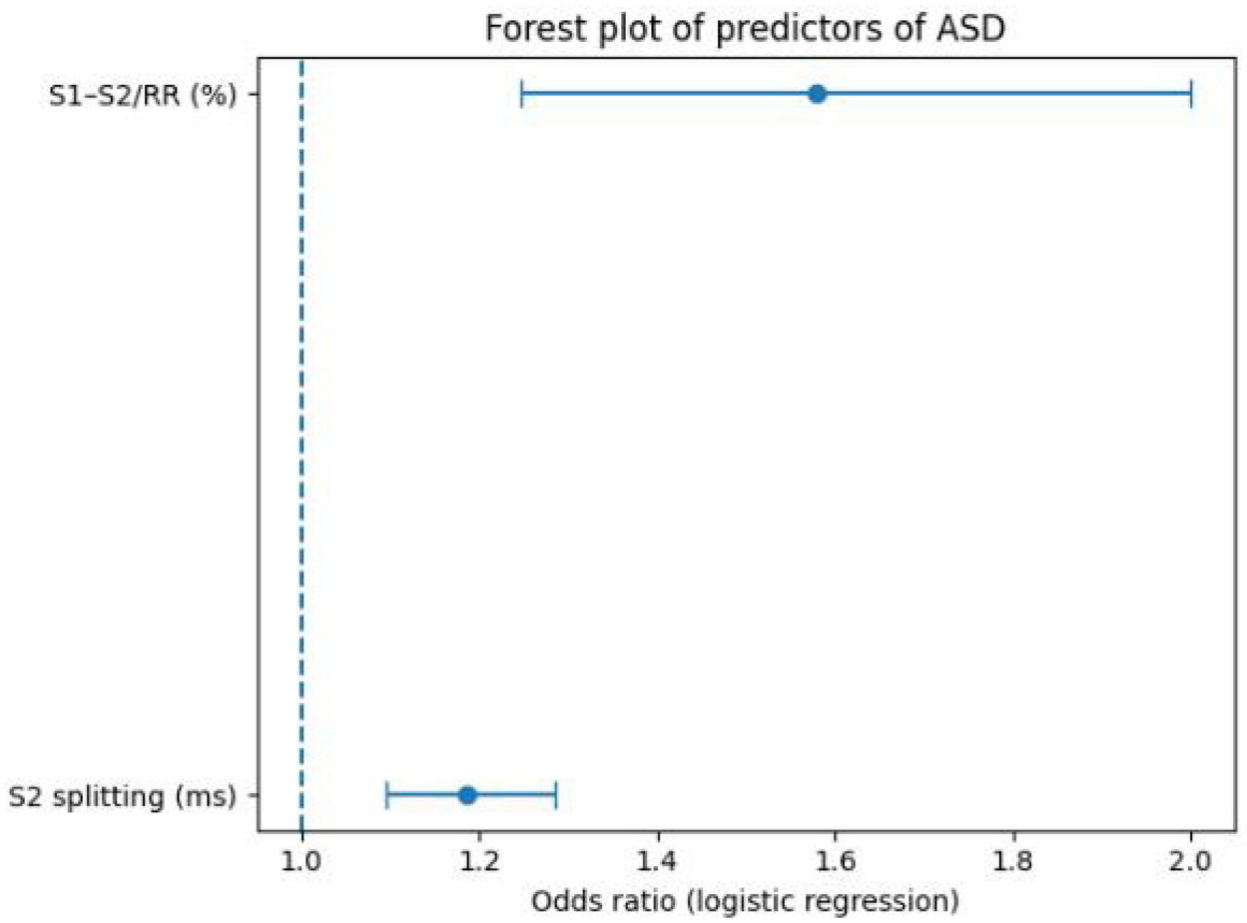
Forest plot of independent predictors of atrial septal defect from multivariable logistic regression.

The plot displays odds ratios (ORs) and 95% confidence intervals (CIs) for S2 splitting time (per 1-ms increase) and S1–S2/RR (%) (per 1% increase). Both variables were included simultaneously in the multivariable model. The vertical dashed line indicates OR = 1.0 (no association). Both predictors showed statistically significant associations with ASD (S2 splitting: OR: 1.19, 95% CI: 1.09–1.29, *P* < 0.001; S1–S2/RR: OR: 1.58, 95% CI: 1.25–2.00, *P* < 0.001) (Figure 6).

## 5 Discussion

### 5.1 Comparison with previous studies on wearable devices for pediatric ASD screening

Recent years have witnessed growing research on wearable cardiac devices for the screening and assessment of congenital heart disease in children, with a focus on phonocardiography and ECG-based technologies. Zang et al. (9) developed a wearable ECG-PCG integrated device and reported that it could detect abnormal heart sounds in children with congenital heart disease with an overall accuracy of 78%, but their study did not specifically focus on secundum ASD or quantify S2 splitting time. Tandon et al. (10) reviewed wearable biosensors for congenital heart disease and noted that most existing devices lack pediatric-specific validation and fail to quantify key hemodynamic indices such as S2 splitting. Lv et al. (5) applied artificial intelligence-assisted auscultation to detect congenital heart disease in children, achieving an AUC of 0.81 for ASD screening, but their system relied on handheld auscultation devices rather than wearable patches and did not correct for heart rate using RR intervals.

In comparison with these previous studies, the current work has three key novel contributions: (1) S2 splitting time and RR-corrected S1–S2/RR (%) were quantified as specific indices for pediatric secundum ASD, rather than using general abnormal heart sound detection; (2) Our wearable patch device demonstrated higher diagnostic accuracy for ASD (AUC = 0.831 for S2 splitting) than the AI-assisted auscultation system (AUC = 0.81) and the general ECG-PCG wearable device (accuracy = 78%); (3) We confirmed that these indices are positively correlated with ASD size and RV volume overload, providing a link between acoustic-electrocardiographic measurements and the severity of anatomical and hemodynamic abnormalities. Additionally, unlike most previous studies that included mixed congenital heart disease types, our study focused exclusively on secundum ASD—the most common pediatric congenital heart disease—making the results more specific and clinically applicable for targeted screening.

### 5.2 Pathophysiological rationale for abnormal acoustic–electrocardiographic indices in ASD

In this cross-sectional study of children aged 4–12 years, we demonstrated that a wearable patch-type device for synchronized phonocardiogram–ECG recording can provide quantitative acoustic–electrocardiographic indices that differentiate secundum ASD from structurally normal hearts. Among the parameters evaluated, S2 splitting time and the RR-corrected systolic fraction S1–S2/RR (%) were significantly increased in ASD compared with controls and showed good to acceptable diagnostic performance, whereas absolute S1–S2 duration and EMAT-derived indices did not differ significantly between groups. ROC analysis identified clinically interpretable cut-off values for S2 splitting time (≥31.75 ms) and S1–S2/RR (%) (≥35.25%), and multivariable logistic regression confirmed both measures as independent predictors of ASD. These findings highlight the potential role of simple heart sound–ECG–based metrics, acquired with a wearable sensor, for non-invasive screening of ASD in children.

From a pathophysiological perspective, the observed prolongation of S2 splitting in the ASD group is consistent with the classic hemodynamic consequences of a left-to-right interatrial shunt. Secundum ASD leads to chronic right atrial and right ventricular volume overload, with increased pulmonary blood flow and delayed right ventricular ejection (12). As a result, the pulmonary component of S2 (P2) occurs later relative to the aortic component (A2), producing a wide and relatively fixed splitting of S2 that shows minimal respiratory variation. This characteristic heart sound finding is well recognized in bedside examination but has rarely been quantified in a standardized, digital manner in pediatric cohorts (13). Our data show that, even under controlled conditions at end-expiration, S2 splitting time measured in milliseconds is substantially longer in ASD than in healthy children, and that this objective marker can be captured reliably using a wearable acoustic–electrocardiographic sensor.

The increase in S1–S2/RR (%) observed in the ASD group further supports the notion of altered systolic timing in these children. The S1–S2 interval reflects the duration of mechanical systole, while normalization to the RR interval provides a heart-rate–corrected measure of the fraction of the cardiac cycle occupied by systole. In our study, absolute S1–S2 did not differ significantly between groups, but the RR-corrected fraction S1–S2/RR (%) was higher in ASD, indicating that, relative to the total cycle length, systole occupied a larger proportion of the cardiac cycle in ASD children than in healthy controls. This pattern is compatible with right ventricular volume loading and prolonged ejection in ASD, even in the absence of overt left ventricular systolic dysfunction. The lack of significant differences in EMAT and EMAT/RR (%) between groups is also noteworthy. EMAT is mainly influenced by left ventricular electromechanical activation and isovolumic contraction; the similar EMAT values in ASD and controls suggest that left ventricular systolic performance was preserved in our cohort, consistent with the inclusion of hemodynamically compensated children without overt cardiomyopathy or significant left-sided disease.

The diagnostic performance metrics reinforce the potential clinical utility of these indices. S2 splitting time achieved an AUC of 0.831, which is generally considered indicative of good discrimination, with high sensitivity (0.90) and moderate specificity (0.66) at the optimal cut-off. Such a profile is well suited to a screening context, where minimizing missed cases is prioritized and positive findings can be confirmed by echocardiography. S1–S2/RR (%) demonstrated a somewhat lower AUC of 0.776 but still provided acceptable discrimination, and its optimal cut-off yielded a sensitivity of 0.88 and specificity of 0.60. Together with the logistic regression results—showing that each 1-ms increase in S2 splitting time and each 1% increase in S1–S2/RR were associated with 19% and 58% higher odds of ASD, respectively—these findings indicate that the two parameters offer complementary and independent information. In practice, a simple combination of S2 splitting time and S1–S2/RR (%) derived from a short wearable recording could be used to flag children at elevated likelihood of ASD for further echocardiographic evaluation.

Clinically, these results suggest several important implications. First, they provide quantitative confirmation of the long-held qualitative observation that wide, relatively fixed S2 splitting is a hallmark of secundum ASD in children. Second, they demonstrate that this information can be captured by a compact, non-invasive, and child-friendly wearable device, rather than relying solely on expert auscultation. Third, the identification of specific numerical thresholds for S2 splitting and S1–S2/RR (%) offers a basis for developing algorithm-driven screening tools that can be deployed in primary care clinics, school health programs, or remote areas where pediatric cardiologists and echocardiography are not readily available (14). By transforming subjective auscultatory findings into objective, reproducible indices, wearable acoustic–electrocardiographic monitoring has the potential to reduce underdiagnosis and delayed recognition of ASD in routine pediatric practice (15).

This study also has several limitations that should be acknowledged. It was conducted at a single center with a relatively modest sample size, which may limit the generalizability of the findings. Only children aged 4–12 years with secundum ASD were included, and results may not be applicable to younger infants, older adolescents, or those with other forms of atrial septal defects or complex congenital heart disease. We did not stratify ASD patients by defect size, shunt magnitude, or presence of mild pulmonary hypertension, factors that could influence acoustic–electrocardiographic indices (16). Recordings were obtained under controlled conditions at end-expiration, which may not fully reflect performance under more variable real-world conditions. Additionally, the detection algorithms and signal processing steps are device- and software-specific, and we did not conduct inter-device or inter-algorithm comparisons. Finally, the cross-sectional design precludes assessment of longitudinal changes in these parameters after transcatheter or surgical closure, and there was no external validation cohort to test the robustness of the identified cut-offs and logistic model (17).

Future work should include multicenter studies with larger and more diverse pediatric cohorts, as well as prospective screening in community or school settings to assess the feasibility and cost-effectiveness of wearable acoustic–electrocardiographic monitoring. Longitudinal follow-up before and after ASD closure is needed to determine how S2 splitting time and S1–S2/RR (%) evolve and whether they have prognostic value. In parallel, refining signal-processing algorithms and integrating these indices into multiparametric or machine-learning models may further enhance diagnostic performance and facilitate broader clinical implementation (18).

## 6 Conclusion

In summary, this cross-sectional study shows that a wearable patch-type phonocardiogram–ECG sensor can quantify acoustic–electrocardiographic indices in children, and that prolonged S2 splitting time and increased S1–S2/RR (%) are characteristic of secundum ASD with good discriminatory ability. These simple, non-invasive metrics may provide useful adjuncts for pediatric ASD screening, particularly where echocardiography is less accessible, but require validation in larger, multicenter cohorts before routine clinical adoption.

## Data Availability

The datasets presented in this study can be found in online repositories. The names of the repository/repositories and accession number(s) can be found in the article/Supplementary Material.
